# PReS-FINAL-2344: Radiographic evaluation of joint space width compared to cartilage thickness as assessed by ultrasonography in knees of children with juvenile idiopathic arthritis

**DOI:** 10.1186/1546-0096-11-S2-P334

**Published:** 2013-12-05

**Authors:** D Pradsgaard, A Hørlyck, AH Spannow, CW Heuck, T Herlin

**Affiliations:** 1 Pediatrics, Aarhus University Hospital Skejby, Aarhus, Denmark; 2 Diagnostic Radiology, Aarhus University Hospital Skejby, Aarhus, Denmark

## Introduction

Joint space narrowing (JSN) is a measureable outcome of the tissue degeneration caused by the inflammatory process of the synovium in arthritis. JSN is usually assessed by conventional radiography. Ultrasonographic measurements of joint cartilage thickness in large and small joints have been validated in a study of healthy children and recently, measurements of distal femoral cartilage of the knee joint have been validated in a group of JIA patients.

## Objectives

To correlate measures of cartilage thickness in the knee assessed by US to the measures of joint space width (JSW) assessed by computerized radiography in children with JIA.

## Methods

Seventy-four children with JIA, aged between 5 and 15 years (median 11.3 yrs), 54 girls and 20 boys were included in the study. One hundred forty-eight knee joints were clinically assessed with regard to swelling within the joint and limitation in the range of movement with pain or tenderness. US assessed distal femoral cartilage thickness and radiography assessed joint space width (JSW) in the femoro-tibial joint space in four spots: medial and lateral femoral condylar areas in both right and left knee. Dijkstra Composite scoring system was used for the radiographical evaluation to describe the inflammatory activity and damage.

## Results

We found a high level of agreement between US and radiographic measures of cartilage thickness and JSW with Rho values between r = 0.52 to 0.81 (p < 0.05 for all four assessed sites). When comparing knees previously affected by joint activity to joints never affected by arthritis, we found no significant difference by US, but we did with radiography.

## Conclusion

US measurements of distal femoral cartilage thickness are well correlated to radiographic measurements of JSW in knees of children with JIA. However, the loss of information by not assessing the tibial cartilage by US may limit the use of US as a tool in the assessment of JSN.

## Disclosure of interest

None declared.

